# Plasma aryl hydrocarbon receptor associated with epicardial adipose tissue in men: a cross-sectional study

**DOI:** 10.1186/s13098-023-01166-y

**Published:** 2023-09-25

**Authors:** Yu-Cheng Cheng, Wei-Chun Ma, Yu-Hsuan Li, Junyi Wu, Kae-Woei Liang, Wen-Jane Lee, Hsiu-Chen Liu, Wayne Huey-Herng Sheu, I-Te Lee

**Affiliations:** 1 Division of Endocrinology and Metabolism, Department of Internal Medicine, Taichung Veterans General Hospital, No. 1650 Taiwan Boulevard, Sect. 4, Taichung, 40705 Taiwan; 2 Institute of Biomedical Sciences, National Chung Hsing University, Taichung, 40227 Taiwan; 3 School of Medicine, National Yang Ming Chiao Tung University, Taipei, 11221 Taiwan; 4 Division of Endocrinology and Metabolism, Department of Internal Medicine, Feng Yuan Hospital, Ministry of Health and Welfare, Taichung, 42055 Taiwan; 5 Department of Computer Science & Information Engineering, National Taiwan University, Taipei, 10617 Taiwan; 6 Cardiovascular Center, Taichung Veterans General Hospital, Taichung, Taiwan; 7 Department of Post-Baccalaureate Medicine, School of Medicine, National Chung Hsing University, Taichung, 402204 Taiwan; 8 Department of Medical Research, Taichung Veterans General Hospital, Taichung, 40705 Taiwan; 9 Department of Nursing, Taichung Veterans General Hospital, Taichung, 40705 Taiwan; 10 National Health Research Institutes, Miaoli County, 35053 Taiwan; 11 School of Medicine, Chung Shan Medical University, Taichung, 40201 Taiwan

**Keywords:** Adipose, Aryl hydrocarbon receptor, Body mass index, Epicardial, Obesity

## Abstract

**Background:**

Epicardial adipose tissue (EAT) is a type of ectopic fat with endocrine and paracrine functions. Aryl hydrocarbon receptor (AhR) is a ligand-activated transcription factor that responds to environmental stimuli. AhR expression is associated with obesity. In this cross-sectional study, we aimed to determine the relationship between circulating AhR concentrations and EAT.

**Methods:**

A total of 30 men with obesity and 23 age-matched men as healthy controls were enrolled. Plasma AhR concentrations were determined at fasting. The EAT thickness was measured on the free wall of the right ventricle from the basal short-axis plane by magnetic resonance imaging.

**Results:**

The participants with obesity had a higher plasma AhR level than the controls (81.0 ± 24.5 vs. 65.1 ± 16.4 pg/mL, P = 0.010). The plasma AhR level was positively correlated with EAT thickness (correlation coefficient = 0.380, P = 0.005). After adjusting for fasting glucose levels, plasma AhR levels were still significantly associated with EAT thickness (95% CI 0.458‒5.357, P = 0.021) but not with body mass index (P = 0.168).

**Conclusion:**

Plasma AhR concentrations were positively correlated with EAT thickness on the free wall of the right ventricle in men. Further investigations are needed to evaluate the causal effects and underlying mechanisms between AhR and EAT.

## Introduction

There has been an increasing trend in body mass index (BMI) in recent decades, and obesity has become a health burden worldwide [1, 2]. The global mean BMI of adult males increased from 21.7 kg/m^2^ in 1975 to 24.2 kg/m^2^ in 2014, and the global proportion of adult males with a BMI ≥ 30 kg/m^2^ increased from 3.2% in 1975 to 10.8% in 2014 [3]. According to the Nutrition and Health Survey in Taiwan, the prevalence of adult obesity, defined as a BMI ≥ 27 kg/m^2^, sharply increased from 11.8% during 1993–1996 to 22.0% during 2013–2014 [4]. Compared to a BMI between 22 and 25 kg/m^2^, a BMI ≥ 30 kg/m^2^ is associated with an increased risk of mortality [5]. In addition to BMI, central obesity, measured using waist circumference, has an independent risk of mortality [6, 7]. Liu et al. [8] reported that waist circumference as well as BMI is important in predicting mortality in Chinese males with prediabetes. However, McNeely et al. [9] reported that the area of visceral fat at the level of the umbilicus was not a better predictor of mortality than BMI.

Epicardial adipose tissue (EAT) is the ectopic fat located between the myocardium and the visceral layer of the pericardium [10, 11]. EAT, as an endocrine and paracrine organ, physiologically exhibits metabolic, thermogenic, and cardioprotective characteristics [12]. However, excessive EAT thickness is associated with metabolic syndrome [13], insulin resistance [14], coronary artery disease [15], and changes in cardiac morphology [16, 17]. EAT is an independent risk factor for both coronary calcification and coronary atheromatous plaque; EAT may more directly reflect the presence of coronary artery disease than the area of abdominal visceral fat [18]. Therefore, EAT is an important risk factor for coronary heart disease [19, 20].

The aryl hydrocarbon receptor (AhR) is a ligand-activated transcription factor of the basic-helix-loop-helix family with a Per-ARNT-Sim domain for regulating downstream responses to environmental stimulation [21]. One of the most important AhR ligands is the environmental pollutant dioxin (2,3,7,8-tetrachlorodibenzo-p-dioxin, TCDD) [22]. The AhR complex (AhR/ligand/heat shock protein 90) is activated after ligand binding and translocates into the nucleus. The AhR/ligand complex can bind to the AhR nuclear translocator (ARNT) and trigger the promoters of target genes, including the cytochrome P450 (CYP) enzymes CYP1A1 and CYP1B1 [23]. Furthermore, the AhR complex also activates mitogen-activated protein kinases (MAPKs), leading to the activation of nuclear factor-kappaB and associated proinflammatory cytokines [24]. Circulating AhR concentrations are higher in subjects with overweight or obesity than in those with normal weight [25]. A high-fat diet can induce vascular AhR protein expression in mice, and inhibition of AhR can attenuate vascular dysfunction induced by a high-fat diet [26].

Activation of the AhR pathway is associated with myocardial ischemia–reperfusion injury through the regulation of mitochondrial oxidative stress and apoptosis [27]. The use of an AhR antagonist can attenuate myocardial injury in the rat myocardial ischemia–reperfusion model [28]. Vilahur et al. [29] also reported that ischemic postconditioning can induce a downregulation of the AhR pathway and reduce reperfusion-induced cell damage to improve cardiac function in pigs after myocardial infarction. Recently, Christensen et al. [30] reported that EAT thickness on the free wall of the right ventricle could significantly predict cardiovascular events and mortality in men. Both EAT thickness and AhR expression have a close relationship with obesity. However, the association between EAT thickness and circulating AhR levels has not been understood. We hypothesize that EAT thickness is positively correlated with circulating AhR levels. Therefore, we conducted a cross-sectional study to assess the EAT thickness and plasma AhR levels in men with and without obesity.

## Materials and methods

### Study design and subjects

This cross-sectional study was conducted at Taichung Veterans General Hospital, Taiwan. The inclusion criteria for study candidates were (1) male Han Chinese adults, (2) having metabolic syndrome defined by the International Diabetes Federation [31], and (3) having obesity defined by a BMI ≥ 27 kg/m^2^ [32]. The exclusion criteria were (1) a known medical history of diabetes mellitus, (2) a known endocrine disease, (3) a known psychological disorder, (4) severe systemic diseases including malignancies, established ischemic heart disease, or immune disorders, and (5) current use of medications that change body weight, including systemic steroids. In addition, we enrolled age-matched men with a BMI < 24 kg/m^2^ as healthy controls.

### Procedures

After enrollment, anthropometric characteristics were measured in the morning after an overnight fast. The measurements of body height (Pharmacia Taiwan Inc., Taipei, Taiwan) and body weight (Detecto, Cardinal Scale Manufacturing Co., Webb City, MO, USA) were performed after participants removed their shoes and any heavy clothing. Waist circumference (kp-1508, King Life, Taipei, Taiwan) was measured at the level of the umbilicus after expiration with the participant breathing quietly and regularly. Blood pressure was detected using the Dinamap^™^ Vital Signs Monitor (Model 1846 SX/P, Critikon, Tampa, FL) after subjects had been sitting at rest for 10 min.

### Biochemical assessments

Blood samples were collected during fasting. Serum samples were prepared for detecting levels of creatinine, total cholesterol, high-density lipoprotein (HDL) cholesterol, and triglycerides. Plasma samples were prepared for detecting levels of glucose, insulin, and AhR. A 75 g oral glucose tolerance test was performed to exclude patients with a diagnosis of diabetes mellitus based on plasma glucose levels ≥ 126 mg/dL at fasting or ≥ 200 mg/dL at 120 min [33].

Concentrations of cholesterol, triglycerides, and creatinine were measured by using commercial kits (Beckman Coulter, Fullerton, USA). Glucose levels were measured using an oxidase–peroxidase method (Wako Diagnostics, Tokyo, Japan). Insulin levels were measured using a commercial kit (Roche Diagnostics GmbH, Mannheim, Germany). AhR levels were determined using the quantitative sandwich enzyme immunoassay with a commercial kit (Cusabio, Wuhan, China). The coefficient of variability (CV) of intra-assay precision for AhR was < 8% based on twenty testes on one plate and the inter-assay CV was < 10%.

The homeostasis model assessment of insulin resistance (HOMA-IR) index was calculated using the following equation: fasting insulin (µIU/mL) × fasting glucose (mmol/L)/22.5 [34]. The estimated glomerular filtration rate (eGFR) was calculated according to the Chronic Kidney Disease Epidemiology Collaboration (CKD-EPI) equation as follows: 141 × (serum creatinine [mg/dL]/0.9)^−0.411^ × 0.993^age (years)^ if the serum creatinine level is ≤ 0.9 mg/dL or 141 × (serum creatinine [mg/dL]/0.9)^−1.209^ × 0.993^age (years)^ if the serum creatinine level is > 0.9 mg/dL [35].

### Magnetic resonance imaging (MRI) assessments

The parameters of the adipose component were assessed by MRI (Siemens Medical Systems, Iselin, New Jersey, USA), as reported in our previous study [36]. Briefly, EAT thickness was measured on the free wall of the right ventricle from the basal short-axis plane [37]. The average thickness was recorded using measurements at three equally spaced points (25%, 50%, and 75% of the full length) along the right ventricular free wall [38]. Participants were asked to hold their breath after expiration while cross-sectional images were taken. Images were transferred to a Siemens Leonardo workstation and the adipose tissue area was calculated using software (Leonardo, Siemens Healthcare, Germany) [39].

### Statistical analysis

All continuous data are presented as the mean ± standard deviation, and categorical data are presented as numbers (percentages). The chi-square test was used to detect significant differences in categorical variables. The data distributions of plasma AhR levels were examined using the Kolmogorov–Smirnov test and were compatible with a normal distribution in both the obesity and control groups (P = 0.128 and 0.106, respectively). An independent sample t test was conducted to detect significant differences in variables between the two groups. The correlation coefficient (*r*) was estimated using Pearson’s correlation test. Linear regression analysis was used to analyze the association between clinical factors and plasma AhR levels. Fasting glucose was used as the variable for glucose dysregulation in the regression analysis because of strong multicollinearity between fasting glucose, fasting insulin, and HOMA IR. A two-sided P value < 0.05 was considered statistically significant. Statistical analyses were performed using SPSS 22.0 (IBM, Armonk, NY, USA).

## Results

**Fig. 1:**
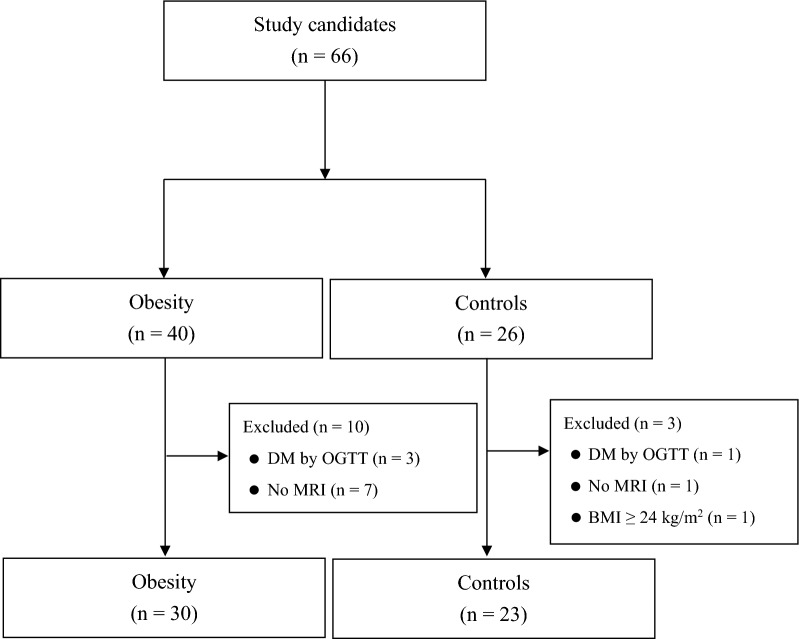
Flow diagram of the enrollment of the study participants. *BMI* body mass index, *DM* diabetes mellitus, *MRI* magnetic resonance imaging, *OGTT* 75 g oral glucose tolerance test

A total of 66 candidates were enrolled in this study, including 40 men in the obesity group and 26 men in the control group. Ten subjects in the obesity group and three subjects in the control group were excluded from the analyses after assessment (Fig. 1).

The characteristics of the 53 participants enrolled in the data analyses are shown in Table 1. There was no significant difference in age between the obesity and control groups (43 ± 11 vs. 39 ± 11 years, P = 0.184). The participants with obesity had a higher body weight (97.8 ± 13.5 vs. 67.1 ± 7.1 kg, P < 0.001), BMI (33.7 ± 4.2 vs. 22.5 ± 1.6 kg/m^2^, P < 0.001), waist circumference (109.3 ± 9.6 vs. 82.1 ± 6.4 cm, P < 0.001), and EAT thickness (6.8 ± 1.8 vs. 3.2 ± 1.4 mm, P < 0.001) than the controls. The participants with obesity also had higher blood pressures (systolic: 135 ± 18 vs. 115 ± 10 mmHg, P < 0.001; diastolic: 79 ± 13 vs. 69 ± 7 mmHg, P = 0.001; respectively), higher fasting glucose levels (5.5 ± 0.5 vs. 5.1 ± 0.4 mmol/L, P = 0.005), higher fasting insulin levels (18.4 ± 11.6 vs. 6.3 ± 2.6 µIU/mL, P < 0.001), higher HOMA-IR (4.5 ± 3.0 vs. 1.4 ± 0.7, P < 0.001), higher fasting triglyceride levels (2.2 ± 0.8 vs. 1.0 ± 0.3 mmol/L, P < 0.001), and lower HDL cholesterol levels (1.1 ± 0.2 vs. 1.5 ± 0.3, P < 0.001) than the controls. However, there was no significant difference in the proportion of current smokers, total cholesterol levels, and the eGFR between these two groups (P > 0.05 for all the variables).

**Table 1:** Clinical data of the subjects with obesity and healthy controls

	Obesity (n = 30)	Healthy controls (n = 23)	P
Age (year)	43 ± 11	39 ± 11	0.184
Body weight (kg)	97.8 ± 13.5	67.1 ± 7.1	< 0.001
Current smoker, n (%)	6 (20.0%)	2 (8.7%)	0.441
BMI (kg/m^2^)	33.7 ± 4.2	22.5 ± 1.6	< 0.001
Waist circumference (cm)	109.3 ± 9.6	82.1 ± 6.4	< 0.001
Systolic BP (mmHg)	135 ± 18	115 ± 10	< 0.001
Diastolic BP (mmHg)	79 ± 13	69 ± 7	0.001
Fasting glucose (mmol/L)	5.5 ± 0.5	5.1 ± 0.4	0.005
Fasting insulin (µIU/mL)	18.4 ± 11.6	6.3 ± 2.6	< 0.001
HOMA-IR	4.5 ± 3.0	1.4 ± 0.7	< 0.001
eGFR (mL/min/1.73 m^2^)	109 ± 19	115 ± 12	0.190
Triglycerides (mmol/L)	2.2 ± 0.8	1.0 ± 0.3	< 0.001
Total cholesterol (mmol/L)	5.0 ± 0.7	4.8 ± 0.9	0.363
HDL cholesterol (mmol/L)	1.1 ± 0.2	1.5 ± 0.3	< 0.001
Epicardial adipose tissue thickness (mm)	6.8 ± 1.8	3.2 ± 1.4	< 0.001

*BMI* body mass index, *BP* blood pressure, *eGFR* estimated glomerular filtration rate, *HDL* high-density lipoprotein, *HOMA-IR* homeostasis model assessment-insulin resistance index

**Fig. 2:**
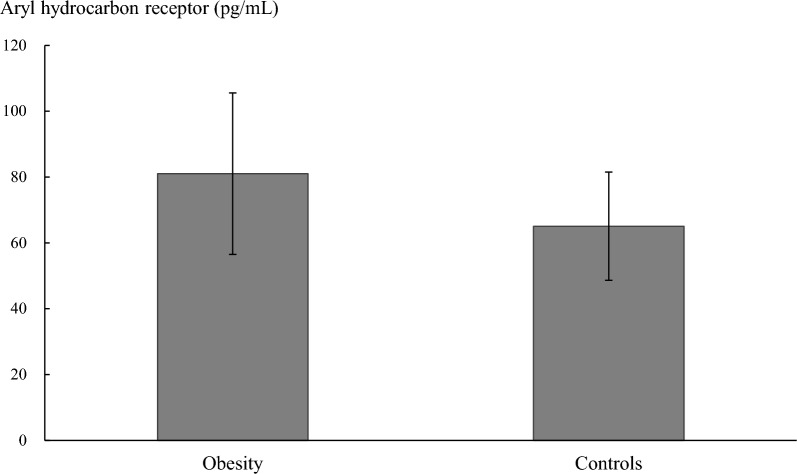
The plasma aryl hydrocarbon receptor concentrations between the obesity and control groups (81.0 ± 24.5 vs. 65.1 ± 16.4 pg/mL, P = 0.010)

**Fig. 3:**
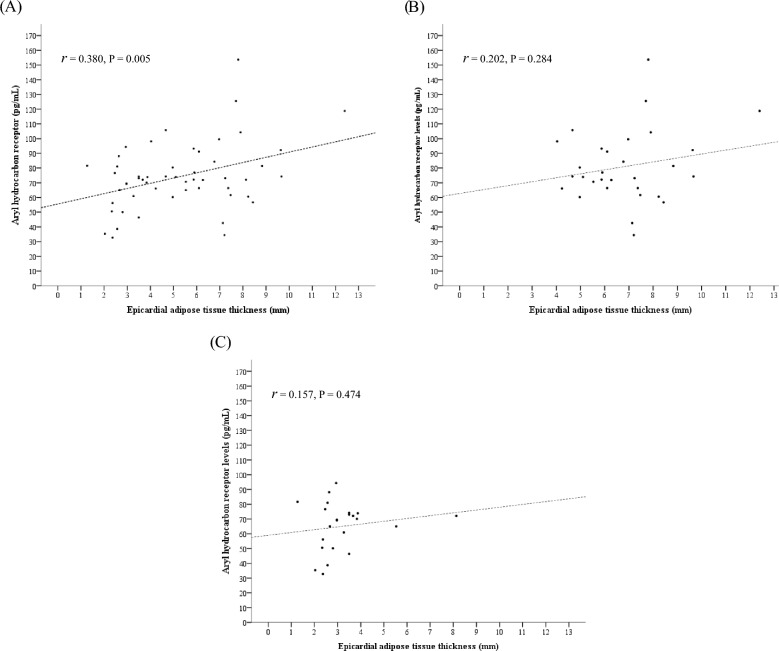
Pearson’s correlation test with the correlation coefficients (*r*) of the epicardial adipose tissue thickness to the plasma aryl hydrocarbon receptor levels in (**A**) all the included subjects, (**B**) the subjects in the obesity group, and (**C**) the subjects in the control group

The participants with obesity had a higher plasma AhR level than the controls (81.0 ± 24.5 vs. 65.1 ± 16.4 pg/mL, P = 0.010; Fig. 2). Furthermore, when the data of all the enrolled participants were analyzed, plasma AhR levels showed significant correlation with the thickness of EAT (*r* = 0.380, P = 0.005; Fig. 3A). However, the correlation between plasma AhR level and EAT thickness was not statistically significant in the obesity group (*r* = 0.202, P = 0.284; Fig. 3B) and in the control group (*r* = 0.157, P = 0.474; Fig. 3C). The univariable regression analysis results showed that plasma AhR levels were significantly associated with EAT thickness, BMI, and fasting glucose levels. The multivariable linear regression analysis results showed that plasma AhR levels were significantly associated with EAT thickness (regression coefficient = 2.907, 95% confidence interval 0.458‒5.357, P = 0.021; Table 2), but were not associated with BMI (P = 0.168) after adjustment with fasting glucose levels.

**Table 2:** Linear regression analysis showing the factors associated with plasma aryl hydrocarbon receptor concentrations (pg/mL)

	Univariable			Mutivariable					
	HR	95% CI	*P*	HR	95% CI	*P*	HR	95% CI	*P*
Age (year)	0.510	(–0.040, 1.060)	0.069						
Body weight (kg)	0.269	(–0.059, 0.596)	0.106						
BMI (kg/m^2^)	0.964	(0.028, 1.901)	0.044				0.670	(–0.293, 1.633)	0.168
Waist circumference (cm)	0.373	(–0.013, 0.759)	0.058						
Systolic BP (mmHg)	0.210	(–0.137, 0.558)	0.230						
Diastolic BP (mmHg)	0.216	(–0.335, 0.766)	0.435						
Fasting glucose (mmol/L)	14.347	(2.755, 25.939)	0.016	10.592	(–0.948, 22.133)	0.071	11.670	(–0.447, 23.788)	0.059
eGFR (mL/min/1.73 m^2^)	− 0.076	(–0.456, 0.305)	0.691						
Triglycerides (mmol/L)	2.729	(–4.707, 10.166)	0.465						
Total cholesterol (mmol/L)	− 2.298	(–10.416, 5.820)	0.572						
HDL cholesterol (mmol/L)	− 14.278	(–33.935, 5.379)	0.151						
Epicardial adipose tissue thickness (mm)	3.523	(1.115, 5.932)	0.005	2.907	(0.458, 5.357)	0.021			

*B* linear regression coefficient, *BMI* body mass index, *BP* blood pressure, *CI* confidence interval, *eGFR* estimated glomerular filtration rate, *HDL* high-density lipoprotein

## Discussion

The main finding of the present study was that plasma AhR concentrations were positively correlated with the thickness of EAT on the free wall of the right ventricle in men. In the linear regression analyses, plasma AhR levels showed a better correlation with EAT thickness than the correlation with BMI or waist circumference. AhR overexpression is associated with obesity and obesity-related inflammation. Rojas et al. [40] reported that visceral adipocytes detected using fluorescence microscopy significantly decreased after AhR antagonist treatment in male mice after high-fat diet feeding, but subcutaneous adipocytes did not significantly respond to AhR antagonist treatment. Similarly, Xu et al. [41] reported that visceral fat, presented as the weight of epididymal white adipose tissue, significantly decreased in AhR-deficient mice compared with wild-type mice after high-fat diet feeding. Moreover, glucose levels, insulin resistance, and inflammatory cytokines were also lower in AhR-deficient mice than in wild-type mice after high-fat diet feeding. Notably, high-fat diet increased the expression of AhR protein in the aorta of male mice [26], but decreased the expression of AhR protein in the liver tissues [42]. Therefore, high-fat diet may enhance the circulating levels of AhR by increasing the vascular AhR protein, but may not increase the AhR protein in the other tissues. Serum AhR levels have been reported to be higher in patients with a BMI ≥ 25 kg/m^2^ than in those with a BMI < 25 kg/m^2^ [25], and Wang et al. [43] also reported that serum AhR levels were inversely correlated with β-cell function presented as an insulinogenic index. The strength of our study is that EAT thickness was shown to have a better correlation with plasma AhR levels than BMI and waist circumference.

AhR plays an important role in adipocyte differentiation, and activation of AhR can significantly promote peroxisome proliferator-activated receptor γ (PPARγ) decay, which was shown to be involved in the mechanism of proteasome-dependent degradation in an in vitro study [44]. AhR activation induced not only adipogenesis but also vascular endothelial dysfunction in an in vivo model of male mice [45]. Distel et al. [46] reported that PPARγ agonists could increase lipid turnover and decrease fatty acid release from EAT in an animal model of rats. Atherosclerotic plaque showed an earlier onset and greater severity in dioxin-treated mice than in those without dioxin exposure [47]. AhR expression, presented as mRNA extracted from peripheral blood mononuclear cells, was higher in patients with coronary artery disease than in controls [48]. In a meta-analysis study, dioxin exposure was significantly associated with the mortality risk of ischemic heart disease [49]. Therefore, higher AhR expression might not only increase atherosclerotic risk but also facilitate EAT to release cytokines and fatty acids, causing adverse myocardial remodeling in ischemic cardiomyopathy [50, 51].

AhR expression has been observed in various tissues and cells, including the endothelium [52, 53]. Without ligand binding, AhR is present in an inactive complex in the cytoplasm; after ligand binding, AhR undergoes transformation and shuttles from the cytoplasm into the nucleus, followed by activation of AhR target genes [54]. Beranek et al. [55] reported a positive correlation between serum levels of AhR and CYP1A1, which is one of the most well-known downstream regulators of the AhR signaling pathway. Hu et al. [56] reported that serum levels of AhR and CYP1A1 were significantly increased in patients with atopic dermatitis and the disease severity significantly correlated with AhR expression in the peripheral blood mononuclear cells. Furthermore, Ramos-García et al. reported that high serum AhR levels were associated with Alzheimer’s disease [57]. High circulating AhR levels are also associated with obesity and glucose dysregulation [25, 43]. Therefore, a high circulating AhR level may serve as a biomarker for the excessive activation of the AhR pathway involved in inflammatory mechanism. In the present study, we observed a significantly positive correlation between plasma AhR levels and EAT thickness. Although we could not demonstrate the causal relationship between plasma AhR levels and EAT in this cross-sectional study, the results of our study suggest that plasma AhR levels reflect the EAT thickness which indicates the cardiovascular risk. Previous studies have demonstrated that AhR pathway is a potential therapeutic target for ischemic heart disease [58]. However, further studies are required to assess the pathophysiological mechanisms involving the AhR signaling pathway and excessive EAT.

There are several limitations of the present study. First, in this cross-sectional study, we did not determine the causal relationship between increased circulating AhR levels and EAT accumulation. Second, we did not directly investigate the mechanisms underlying the association between AhR and EAT. Third, we did not assess the source of plasma AhR in the subjects. Fourth, we could not differentiate the phenotypes of epicardial adipocytes based on MRI. Wang et al. [59] reported that a conversion from brown adipose tissue to white adipose tissue can induce the local development of coronary atherosclerosis. Finally, we only enrolled male adults without diabetes mellitus because previous studies reported that the association between EAT cardiovascular risk was stronger in males than in the females [30, 60]. Moreover, plasma AhR levels are higher in the male patients with pancreatic cancer than in the female patients with pancreatic cancer [61]. Furthermore, AhR activation induces different responses in males and females regarding glucose regulation [62]. This study included participants from the Han Chinese ethnicity. Therefore, our findings need to be verified further in other populations.

## Conclusions

Our results demonstrated that plasma AhR concentrations were significantly higher in men with obesity than in those without obesity. In particular, plasma AhR concentrations were positively correlated with the thickness of EAT on the free wall of the right ventricle, and the correlation was better than that with BMI after adjusting for the fasting glucose levels.

## Data Availability

The datasets used and/or analyzed during the current study are available from the corresponding author on reasonable request.

## Abbreviations

AhR: Aryl hydrocarbon receptor
ARNT: Aryl hydrocarbon receptor nuclear translocator
BMI: Body mass index
CKD-EPI: Chronic Kidney Disease Epidemiology Collaboration
CV: Coefficient of variability
CYP: Cytochrome P450
EAT: Epicardial adipose tissue
eGFR: Estimated glomerular filtration rate
HDL: High-density lipoprotein
HOMA-IR: Homeostasis model assessment of insulin resistance
MAPK: Mitogen-activated protein kinase
MRI: Magnetic resonance imaging
PPARγ: Peroxisome proliferator-activated receptor γ
